# Neuroprotective effects of PPARα in retinopathy of type 1 diabetes

**DOI:** 10.1371/journal.pone.0208399

**Published:** 2019-02-04

**Authors:** Elizabeth A. Pearsall, Rui Cheng, Satoshi Matsuzaki, Kelu Zhou, Lexi Ding, Bumsoo Ahn, Michael Kinter, Kenneth M. Humphries, Alexander B. Quiambao, Rafal A. Farjo, Jian-xing Ma

**Affiliations:** 1 Angiogenesis Laboratory, Department of Ophthalmology, Harvard Medical School, Massachusetts Eye and Ear Infirmary, Boston, MA, United States; 2 Department of Physiology, University of Oklahoma Health Sciences Center, Oklahoma City, OK, United States; 3 Aging and Metabolism Research Program, Oklahoma Medical Research Foundation, Oklahoma City, OK, United States; 4 Department of Biochemistry, University of Oklahoma Health Sciences Center, Oklahoma City, OK, United States; 5 EyeCRO LLC, Oklahoma City, OK, United States; 6 Harold Hamm Oklahoma Diabetes Center, Oklahoma City, OK, United States; Wayne State University, UNITED STATES

## Abstract

Diabetic retinopathy (DR) is a common neurovascular complication of type 1 diabetes. Current therapeutics target neovascularization characteristic of end-stage disease, but are associated with significant adverse effects. Targeting early events of DR such as neurodegeneration may lead to safer and more effective approaches to treatment. Two independent prospective clinical trials unexpectedly identified that the PPARα agonist fenofibrate had unprecedented therapeutic effects in DR, but gave little insight into the physiological and molecular mechanisms of action. The objective of the present study was to evaluate potential neuroprotective effects of PPARα in DR, and subsequently to identify the responsible mechanism of action. Here we reveal that activation of PPARα had a robust protective effect on retinal function as shown by Optokinetic tracking in a rat model of type 1 diabetes, and also decreased retinal cell death, as demonstrated by a DNA fragmentation ELISA. Further, PPARα ablation exacerbated diabetes-induced decline of visual function as demonstrated by ERG analysis. We further found that PPARα improved mitochondrial efficiency in DR, and decreased ROS production and cell death in cultured retinal neurons. Oxidative stress biomarkers were elevated in diabetic *Pparα*^*-/-*^ mice, suggesting increased oxidative stress. Mitochondrially mediated apoptosis and oxidative stress secondary to mitochondrial dysfunction contribute to neurodegeneration in DR. Taken together, these findings identify a robust neuroprotective effect for PPARα in DR, which may be due to improved mitochondrial function and subsequent alleviation of energetic deficits, oxidative stress and mitochondrially mediated apoptosis.

## Introduction

Diabetic retinopathy (DR) is a common microvascular complication of diabetes, and is the leading cause of blindness in the working-age population [1]. DR is considered to be a microvascular complication, and current therapeutic approaches target retinal edema and the neovascular lesions characteristic of advanced disease [1]. However, retinal neurodegeneration precedes clinically overt microvascular pathologies, and a growing body of evidence suggests that neurodegeneration contributes to the development of microvascular dysfunction and neovascularization [2]. Neuroprotective therapies are therefore being investigated as potential modalities for DR [3].

Two independent perspective clinical trials demonstrated unexpectedly that fenofibrate, a PPARα agonist used to treat dyslipidemia, had unprecedented therapeutic effects in DR [4, 5]. However, this was identified as a tertiary outcome by intention-to-treat analysis in both trials, so these unexpected findings gave little insight into the physiological and molecular mechanisms of action. Fenofibrate and PPARα have since been a topic of intense investigation in DR, although prior studies have focused predominately upon microvascular pathologies of DR [6–9]. One prior study identified that fenofibrate was neuroprotective in retinopathy of type 2 diabetes, but did not determine whether these effects were related to PPARα activation or evaluate the molecular mechanism of action [10]. In this study, we sought to determine if PPARα is also neuroprotective in retinopathy of type 1 diabetes using both functional and biochemical analyses in rats treated with fenofibrate, and in diabetic *Pparα*^*-/-*^ mice. We identified for the first time that PPARα is neuroprotective in retinopathy of type 1 diabetes, and subsequently sought to identify the molecular basis for this effect.

Phototransduction and transmission of visual signals is energetically demanding, meaning that retinal neurons must consistently produce large amounts of ATP [11]. Retinal mitochondria therefore function at full capacity with a limited reserve, and have a high oxidative capacity [12]. Therefore, decreased mitochondrial energy efficiency and mitochondrial dysfunction are particularly detrimental to retinal neurons, rapidly leading to neurodegeneration. Mitochondrial dysfunction is thus linked to a litany of retinal diseases, including DR [13].

Mitochondria produce about 95% of cellular ATP through oxidative phosphorylation, and regulate cellular energy metabolism and apoptosis [13]. Mitochondria are also a primary source of intracellular reactive oxygen species (ROS), which are generated when mitochondria are not making ATP consistently and have a high proton motive force, and when the NADH/NAD^+^ ratio is increased [14]. Physiologic levels of ROS are essential for normal cell signaling processes, but when ROS production is pathologically increased, such as through mitochondrial dysfunction in DR, oxidative damage to cellular macromolecules can lead to retinal cell death [15]. Mitochondrial dysfunction in DR may also lead to primary energetic shortages and mitochondrially mediated apoptosis, which could also contribute to neurodegeneration.

The etiology of mitochondrial dysfunction in diabetes remains incompletely understood, but mitochondrial dysfunction plays a causative role in diabetic complications. Decreased mitochondrial efficiency occurring secondary to metabolic inflexibility and posttranslational modification of mitochondrial proteins in diabetes may decrease mitochondrial efficiency and increase ROS production [16]. Subsequent oxidative damage to mitochondrial DNA may further contribute to mitochondrial dysfunction, perpetuating a vicious cycle [15].

PPARα is a well-known regulator of energy metabolism, and prior studies have shown that PPARα increases retinal energy efficiency in pathological conditions [17, 18]. We also recently demonstrated that endogenous PPARα is necessary for retinal lipid metabolism in normal physiological conditions [19]. These effects may be due to regulation of mitochondrial fatty acid oxidation, and potentially of glucose homeostasis [17]. We therefore hypothesized that PPARα may improve mitochondrial function in diabetes, which would alleviate oxidative stress, energetic deficits and mitochondrial damage. To address this hypothesis, we measured retinal electron transport chain activity in diabetic rats treated with the PPARα agonist fenofibrate, and used a targeted proteomics approach to measure oxidative stress biomarkers in diabetic *Pparα*^*-/-*^ mice. Further, we subjected cultured retinal neurons to the diabetic stressor 4-hydroxynonenal (4-HNE), and assessed cytoprotective, antioxidant and mitoprotective effects of PPARα activation and overexpression.

The overarching goal of these studies was to evaluate neuroprotective effects of PPARα in retinopathy of type 1 diabetes, and assess its molecular mechanisms of action. We identified that PPARα was neuroprotective in type 1 DR, and that these effects may be mediated through improved mitochondrial function.

## Materials and methods

### Data availability

All raw data is accessible via the Harvard Dataverse: https://dataverse.harvard.edu/dataset.xhtml?persistentId=doi%3A10.7910%2FDVN%2FITLZ9Z

### Statistics

All experiments in the study compared three or more experimental groups, so we used an ordinary one-way ANOVA with Tukey’s post-hoc comparison to analyze all data. We used graph Pad Prism 7 Software (La Jolla, CA). p≤0.05 was considered to be statistically significant.

### Experimental animals

Brown Norway and Sprague Dawley rats were purchased from Charles River Laboratories (Wilmington, MA). Both wild-type (WT) and PPARα-knockout (*Pparα*^*-/-*^) mice in the C57-BL/6J background were bred in-house. Care, use and treatment of all animals in this study were in strict agreement with the Public Health Service Policy on Care and Use of Laboratory Animals and guidelines in the Care and Use of Laboratory Animals set forth by the University of Oklahoma Institutional Animal Care and Use Committee. All protocols were approved by the IACUC prior to beginning (approval number 14-032-SSHT).

### Selection of diabetic animal models

A well-known limitation of DR research is that different diabetic animal models develop various pathophysiological aspects of DR at different time points, and to different extents [20]. To best assess neuroprotective effects of PPARα in DR of type 1 diabetes, we selected animal models and time points of DR based on previous characterizations of the models, such that we could reasonably expect declines in our control groups.

For visual acuity studies, STZ brown Norway rats were used to evaluate whether PPARα activation preserved visual acuity, as indicated by the optokinetic reflex. We selected this model because diabetic brown Norway rats reliably develop a robust decline in the optokinetic reflex by week 8 of DR [21]. We therefore measured the optokinetic reflex at weeks 8 and 12 of DR. Contrastingly, optokinetic tracking has not been used to assess visual acuity in STZ mice, and cannot be used in albino animals, which lack the optokinetic reflex.

To determine if short-term treatment with a PPARα agonist arrested cell death in DR, we used the Sprague Dawley rat model. Sprague Dawley rats are known to develop robust retinal degeneration beginning at week 2 of DR, as detected by immunohistochemical analyses and DNA fragmentation ELISA [22]. We therefore used 4-week diabetic Sprague Dawley rats treated with fenofibrate for one week prior to the experimental endpoint, and measured total retinal cell death with a DNA fragmentation ELISA. Contrastingly, the DNA fragmentation ELISA has not been used to measure neurodegeneration in STZ mice or brown Norway rats, so the optimal time point for an acute effect in early-stage DR is unknown in these models.

To evaluate direct effects of PPARα, we used *Pparα*^*-/-*^ STZ mice. The neurodegenerative phenotype in STZ mice is not as robust as in STZ rats [20], but neurodegeneration does occur in advanced DR, as previously demonstrated by a diminished electroretinogram (ERG) response [23]. We therefore recorded ERG response at 28 weeks diabetic, and performed mechanistic analyses at this advanced time point.

### Brown Norway rat streptozotocin model of visual acuity

In order to determine if PPARα activation was able to preserve visual acuity in DR, we measured the optokinetic reflex in STZ-injected brown Norway rats using optokinetic tracking. For optokinetic tracking, it is necessary to use pigmented animals, as albino rats are deficient in the optokinetic reflex [24].

AT 10 weeks old, female brown Norway rats were injected intraperitoneally with 45mg/kg STZ or vehicle control. Blood glucose was measured 72 hours after injection and weekly thereafter. Only animals with persistent hyperglycemia (>350 mg/dL) were used as diabetic rats. Weight and blood glucose for brown Norway STZ rats are shown in S1 and S2 Tables.

Rats were fed with control chow or medicated chow containing 0.03% and 0.015% fenofibrate for non-diabetic and diabetic rats, respectively. These percentages were calculated based upon the weight and food consumption of diabetic and non-diabetic rats, and equated to a dosage of 12–14 mg/kg fenofibrate for both groups.

### Sprague Dawley rat streptozotocin model

At 10 weeks old, male Sprague Dawley rats were injected intraperitoneally with 65mg/kg streptozotocin (STZ) dissolved with sodium citrate to induce diabetes, and control non-diabetic rats were injected with vehicle control, as described previously [6]. Blood glucose was measured 72 hours after STZ injection, and weekly thereafter. Only animals with persistent hyperglycemia (>350 mg/dL) were used as diabetic rats. Weight and blood glucose for Sprague Dawley rats are shown in S3 and S4 Tables.

Animals remained diabetic for three weeks, and were then injected with 10 mg/kg/day fenofibric acid (Feno-FA) (AK Scientific; Union City, CA) or vehicle control (DMSO) intraperitoneally for one week prior to the experimental endpoint. Fenofibric acid is the bioactive fenofibrate metabolite, and was used in lieu of fenofibrate for intraperitoneal administration due to improved solubility in aqueous solution. We chose not to use fenofibric acid for dietary studies because the acidity may cause gastritis.

### Mouse streptozotocin model

At 10 weeks old, wild-type (WT) and PPARα-knockout (*PPARα*^*-/-*^) mice in the C57-BL/6J background were injected with 55 mg/kg STZ for 3 consecutive days, and injected with 50 mg/kg STZ for an additional two consecutive days, or with vehicle control for five days as described previously [9]. Blood glucose was measured five days after injection, and monthly for the duration of the experiment. Only mice with persistent hyperglycemia (blood glucose >350 mg/dL) were considered diabetic and included in analysis. Weight and blood glucose for STZ mice are shown in S5 and S6 Tables.

### Optokinetic tracking (OKT)

Optokinetic tracking (OKT) was used to test the visual function of diabetic and non-diabetic brown Norway rats ± fenofibrate using the virtual optokinetic system (OptoMotry system; Cerebral mechanics; Lethbridge, AB, Canada) as described previously [25, 26]. Briefly, rats were placed without movement restriction on a platform at the center of a virtual-reality chamber comprised of four monitors that display sine wave gratings rotating at 12 deg/sec. Rats were monitored through a video camera, which was continuously re-centered on the head of the animal, and the presence or absence of the optokinetic reflex was recorded. To assess visual acuity, the grating began at a spatial frequency of 0.042 cyc/deg, which gradually increased until head turning was no longer observed. Spatial frequency threshold, a measure of visual acuity, was determined automatically with the OKT software, which used a staircase paradigm based upon head-tracking movements. N is expressed as number of animals per treatment group, per timepoint.

### Electroretinograms

Electroretinogram (ERG) recordings were conducted in diabetic and non-diabetic WT and *Pparα*^*-/-*^ mice 24 weeks diabetic as described previously [27]. Briefly, mice were dark-adapted at least 12 hours prior to the procedure, and were then anesthetized with an 85/12 mg/mL ketamine/xylazine mixture (Mylan Institutional; Galway, Ireland and Akorn Inc; Decatur, IL). Pupils were dilated with Atropine (Alcon Laboratories; Ft. Worth, TX) just prior to the procedure. ERG responses were recorded with a platinum needle electrode placed on the corneal surface, a reference electrode placed at the nasal formix, and a ground electrode on the foot. Fourteen responses to a 600 cd·s/m^2^ light stimulus of 10 mSec with flash intervals of 20 sec were recorded. For quantitative analyses, the B-wave amplitude was measured between A-and -B-wave peaks. N is expressed as number of retinas per treatment group.

### DNA fragmentation ELISA

DNA cleavage was measured using an ELISA-based kit (Cell Death Detection ELISA; Roche Applied Science; Indianapolis, IN) as described previously with a few minor modifications [28]. Sample preparation of whole retinal homogenates and ELISA assay were performed as by Zheng et al. [28], and relative fragmentation was expressed as OD (405/490 nm) normalized by RC/DC (Biorad; Hercules, CA) OD (570 nm). N is expressed as the number of retinas per treatment group.

### Cell culture

R28 rod precursor cells were a kind gift of Dr. Gail Seigel [29]. Cells were cultured in Dulbecco’s modified Eagle’s medium (DMEM) containing 1 mM D-glucose, 10% heat-inactivated fetal bovine serum (FBS; Cellgro, Manassas, VA) and 1% antibiotic-antimycotic solution (Cellgro; Manassas, VA). All cells used were between passages 10 and 15 from the original stock.

### Adenovirus preparation

PPARα-expressing adenovirus was generated previously [9]. Preparation, amplification, and titration of the recombinant adenovirus were performed as described previously [30, 31]. Ad-β-Galactosidase prepared previously was used as a control [30].

### MTT assay

Cells were seeded in a 24-well plate and treated as described below. After treatment, a Vybrant MTT Cell Proliferation Assay Kit (Thermofisher; Waltham, MA) was used to quantify cell viability according to manufacturer’s instructions.

#### Fenofibric acid treatment

Cells were seeded at a density of 1.5 x 10^4^ cells/mL in a 24-well plate and allowed to adhere for 24 hours. Cells were then treated with 4-Hydroxynonenyl (4-HNE) (Abcam; Cambridge, MA) and 25 μM Feno-FA for 24 hours, and cell viability quantified as described above.

#### Adenovirus treatment

Cells were seeded at a density of 7.25 x 10^3^ cells/mL in a 24-well plate and allowed to adhere for 24 hours. Cells were then infected with adenovirus expressing β-Galactocidase (Ad-β-Gal) or PPARα (Ad- PPARα) (MOI = 20) described above, and incubated for 24 hours. Cells were then treated with 4-HNE for 24 hours, and cell viability quantified as described above.

### NADH oxidation assay

To quantify mitochondrial NADH-linked respiration, the consumption of exogenously added NADH was measured spectrophotometrically in diabetic and non-diabetic Sprague Dawley rats treated with vehicle or Feno-FA as described previously [32] with minor modifications. Animals were euthanized with pentobarbital, and retinas rapidly excised and snap-frozen in liquid nitrogen. Retinas were homogenized in 500μL 25 mM MOPS/1mM EDTA pH 7.4 and sonicated. Prior to the reaction, retinal homogenates were diluted 1:20 in assay buffer (25 mM MOPS/10mM KCl, pH 7.4). Exogenous NADH (100 μM; Sigma; St. Louis, MO) was added and its oxidation rate monitored spectrophotometrically at 340 nm. Protein concentration of retinal homogenates was measured using a BCA assay (Pierce; Green Island, NY) according to manufacturer’s protocol. Data are expressed as ratio of nmol NADH consumed per minute per mg of total retinal protein. All assays were performed at room temperature. N is expressed as the number of retinas per treatment group.

### Seahorse extracellular flux analysis

To measure mitochondrial function in cultured retinal neurons, we used a Seahorse XFe24 extracellular flux analyzer to measure mitochondrial respiration in cultured retinal neuronal precursor cells treated with the DR-like stressor 4-HNE and treated with fenofibric acid or infected with PPARα-expressing adenovirus.

#### Fenofibric acid treatment

Cells were seeded at a density 1.5 x 10^4^ cells/mL in a 24-well plate in Dulbecco’s modified Eagle’s medium (DMEM) containing 1 g/L D-glucose, 10% heat-inactivated fetal bovine serum (FBS; Cellgro, Manassas, VA) and 1% antibiotic-antimycotic solution (Cellgro; Manassas, VA). Cells were allowed to adhere for 24 hours before treatment. Two hours prior to 4-HNE exposure, cells were treated with vehicle control (DMSO) or 25 μM fenofibric acid. After pre-treatment, cells were exposed to 15 μM 4-HNE for 4 hours prior to analysis.

#### Adenovirus infection

Cells were seeded and cultured as above, and allowed to adhere for 24 hours before adenoviral infection. Cells were then infected with either β-Gal or PPARα-expressing adenovirus for 24 hours. Cells were subsequently exposed to 15 μM 4-HNE for 4 hours prior to analysis.

#### Extracellular flux analysis

Four hours after 4-HNE exposure, oxygen consumption rates were measured using a Seahorse XFe24 Flux Analyzer following the manufacturer’s protocol and as described previously with minor modifications [19]. Prior to analysis, culture media as described above was replaced with basal DMEM. Pyruvate (1 mM) was used as an oxidizable substrate. Oligomycin was injected into port A to a final concentration of 1 μM, FCCP was injected into port B to a final concentration of 2 μM, and Antimycin A (AA) was injected in Port C to a final concentration of 1 μM. Maximal respiration was calculated by subtracting non-mitochondrial respiration from the rate after injection of FCCP. Respiratory reserve was calculated as the relative ratio of basal respiratory rate to maximal respiratory rate. N is expressed as the number of wells per treatment group.

### Fluorescent detection of intracellular ROS

A ROS-sensitive, probe-based method (H_2_DCFDA; Molecular Probes; Eugene, OR, Cat # D399) was used as described previously to detect intracellular ROS [33] in R28 cells. Briefly, cells were treated as described below prior to the assay. Growth medium was then removed, and replaced with serum-free, phenol red-free media. Cells were incubated with 5 μM dye for 30 minutes at 36°C and returned to serum-free, phenol red-free media for 15 minutes before fluorescence was measured per manufacturer’s instructions.

#### Fenofibric acid treatment

Cells were seeded at a density of 1.5 x 10^4^ cells/mL in a 24-well plate and allowed to adhere for 24 hours. Cells were pre-treated with 25 μM Feno-FA for 7 hours, and subsequently treated with 4-HNE for one hour prior to measurement of ROS.

#### Adenovirus treatment

Cells were seeded at a density of 7.25 x 10^3^ cells/mL in a 24-well plate and allowed to adhere for 24 hours. Cells were then infected Ad-β-Gal or Ad- PPARα (MOI = 20), and incubated for 24 hours. Cells were then treated with 4-HNE for one hour prior to measurement of ROS.

### Mass spectrometry

Mass spectrometry was utilized for targeted quantitative proteomic analysis of metabolic pathways in diabetic and non-diabetic WT and *Pparα*^*-/-*^ retinas as described previously with minor modifications [34].

#### Sample preparation

Mice (n = 6 mice/group, 28 weeks diabetic) were euthanized with carbon dioxide asphyxiation and PBS-perfused. Retinas were dissected and snap-frozen in liquid nitrogen. Both retinas from each animal were pooled, homogenized in 200 μL Holt’s lysis buffer and sonicated and then spun 500 xg for 5 min at 4°C, and supernatant was collected. Protein was quantified using a BioRad detergent compatible kit (Hercules, CA) according to manufacturer’s protocol. The volume equivalent to 60 μg protein was taken for analysis, and 8 pmol bovine serum albumin added as a non-endogenous internal standard. Samples were mixed, heated and cooled to room temperature. 1 mL of acetone was added and proteins precipitated overnight at -20°C. The resultant pellet was reconstituted in 6 0μL of sample loading buffer and a 20 μL aliquot resolved by short-run gel electrophoresis (1.5 cm into gel), and gel fixed and stained. Each lane was cut as a single sample, washed, reduced with DTT, alkylated with idoacetamide and digested overnight with trypsin. The peptides from the digestion were extracted, evaporated to dryness and reconstituted in 150 μL 1% acetic acid for analysis.

#### LC-tandem mass spectrometry analysis

An Eskigent nanoflow system (Sciex; Framingham, MA) was used for liquid chromatography (LC) and ThermoScientific TSQ Vantage triple quadrupole was used for mass spectrometry (Thermo Scientific; San Jose, CA). A 75 μM x 11 cm column packed with Phenometrix Jupiter C18 was used. 6 μL aliquots of sample were injected for each analysis. Peptides were eluted with a linear gradient of acetonitrile in water with 0.1% formic acid. For data analysis, the open-source program Skyline (developed by Dr. Michael MacCoss, University of Washington) was used. Total protein responses were calculated as the geometric mean of the abundance of peptides measured for each protein, and normalized to bovine serum albumin (BSA) total protein response. The final abundance of each protein is calculated as pmol/100 μg total protein based on the ratio to BSA and the amount of BSA added, and data expressed as the relative amount of protein in experimental groups relative to control.

## Results

### Neuroprotective effect of PPARα activation in retinopathy of type 1 diabetes

**Fig 1:**
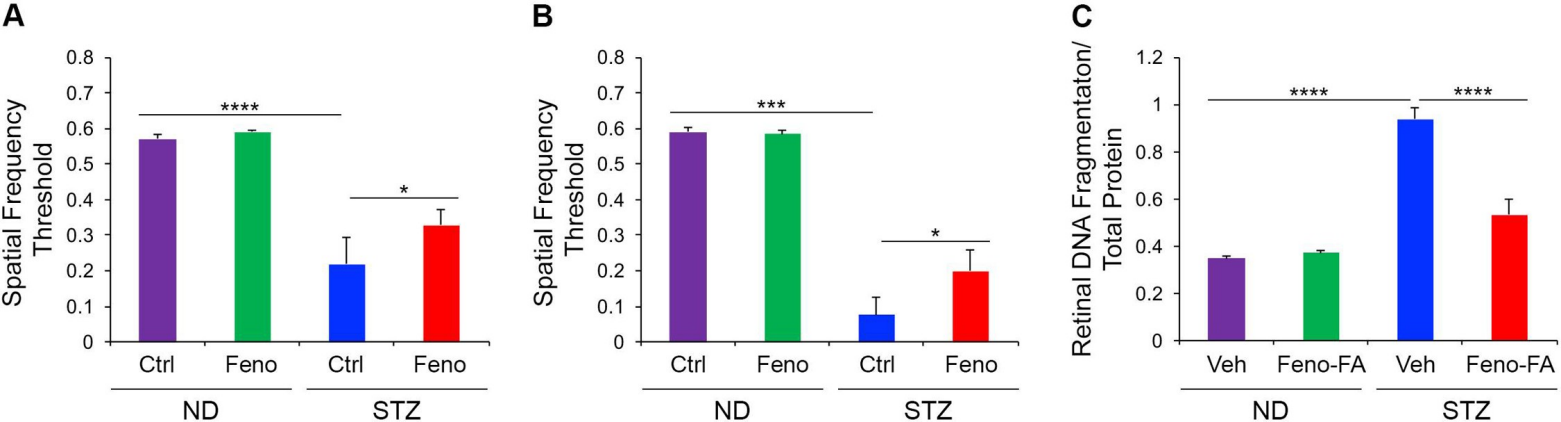
Neuroprotective effects of PPARα activation in diabetic retinopathy. **(A-B)** Optokinetic tracking in streptozotocin (STZ)-induced type 1 diabetic Brown Norway rats revealed that visual function, as detected by spatial frequency threshold, declined at **(A)** 8 weeks and **(B)** 12 weeks diabetic relative to non-diabetic (ND) rats, and was partially restored by oral treatment with PPARα agonist fenofibrate (Feno) (ND-Ctrl n = 6; ND-Feno n = 5; STZ-ctrl n = 8; STZ-Feno n = 7 rats) **(C)** DNA fragmentation ELISA revealed that total retinal apoptosis was increased in diabetic Sprague Dawley rats at 4 weeks diabetic, and was decreased by one week daily intraperitoneal injection of fenofibric acid (Feno-FA) relative to vehicle (Veh, DMSO)-injected rats (ND-Veh n = 13; ND-Feno-FA n = 18; STZ-Veh n = 17; STZ-Feno-FA n = 21 retinas). Data are expressed as mean ±SEM. *p≤0.05; ***p≤0.001, ****p≤0.0001, one-way ANOVA with Tukey’s post-hoc comparison.

We utilized STZ-diabetic rat models, which develop robust retinal neurodegeneration [20], to assess neuroprotective effects of PPARα activation in DR. We used optokinetic tracking to demonstrate that visual acuity, as detected by spatial frequency threshold [21], significantly decreased in diabetic rats and 8 and 12 weeks diabetic (p ≤ 0.001), and was partially restored by continuous oral treatment with PPARα agonist fenofibrate (p ≤ 0.05) **(Fig 1A and 1B)**. Fenofibrate did not affect visual acuity in non-diabetic rats. We next assessed whether short-term PPARα activation could arrest retinal apoptosis in diabetic retinas. We found that total retinal apoptosis, as measured by a DNA fragmentation ELISA, was significantly increased in STZ rats at 4 weeks of diabetic (p ≤ 0.0001), and was decreased by daily intraperitoneal injection of fenofibric acid for one week prior to endpoint (p ≤ 0.0001) **(Fig 1C)**. Fenofibric acid did not affect retinal apoptosis in non-diabetic rats. Together, these findings suggested that long-term treatment with a PPARα agonist protected retinal function in type 1 diabetes, and that short term treatment alleviated retinal apoptosis.

### Increased retinal neurodegeneration in diabetic *Pparα*^*-/-*^ mice

**Fig 2:**
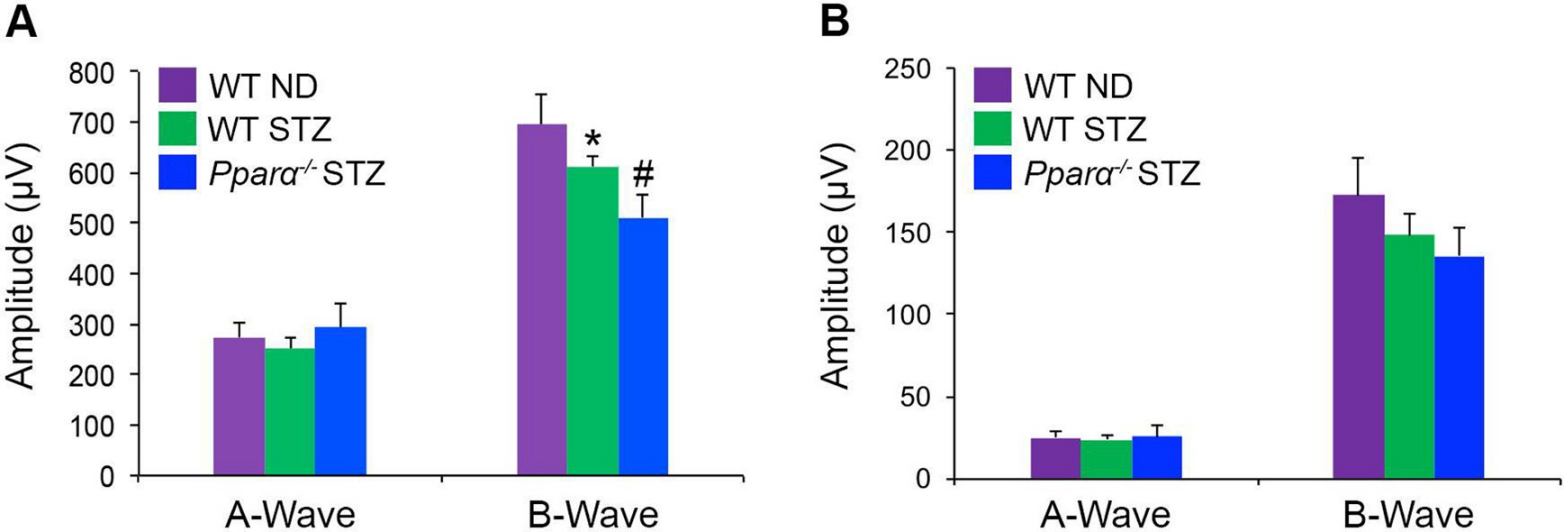
Exacerbated retinal neurodegeneration in diabetic *Pparα*^*-/-*^ mice. **(A-B)** An electroretinogram revealed that **(A)** Scotopic (rod) B-wave declined in wild-type (WT) STZ-diabetic mice relative to non-diabetic (ND) mice at 24 weeks diabetic, and further declined in diabetic *Pparα*^*-/-*^ mice, and **(B)** Photopic (cone) A-Wave and B-wave were not significantly different. (WT ND n = 9; WT STZ n = 10; KO STZ n = 11 retinas). Data are expressed as mean ± SEM. *p≤0.05 relative to WT ND; #p≤0.05 relative to WT diabetic, one-way ANOVA with Tukey’s post-hoc comparison.

To assess the effects of PPARα ablation on retinal neurodegeneration, we utilized the STZ mouse model, which develops subtle neurodegeneration in long-term diabetes [35]. We found that retinal function, as detected by electroretinogram (ERG), was decreased in wild-type STZ mice at 24 weeks diabetic, and further declined in diabetic *Pparα*^*-/-*^mice. Scotopic (rod) A-wave was unaffected by DR, while B-wave declined in wild-type STZ mice (p ≤ 0.05), and further declined in *Pparα*^*-/-*^ STZ mice (p ≤ 0.05) **(Fig 2A)**. Photopic (cone) A-wave and B-wave were not significantly decreased by DR (**Fig 2B**). The murine retina is rod-dominant and diabetic retinal cell death occurs primarily in secondary and tertiary neurons, so these results are consistent with prior observations [20, 36]. Cumulatively, these data suggested that loss of PPARα exacerbated neurodegeneration in DR.

### *In vitro* protective effects of PPARα activation and overexpression

**Fig 3:**
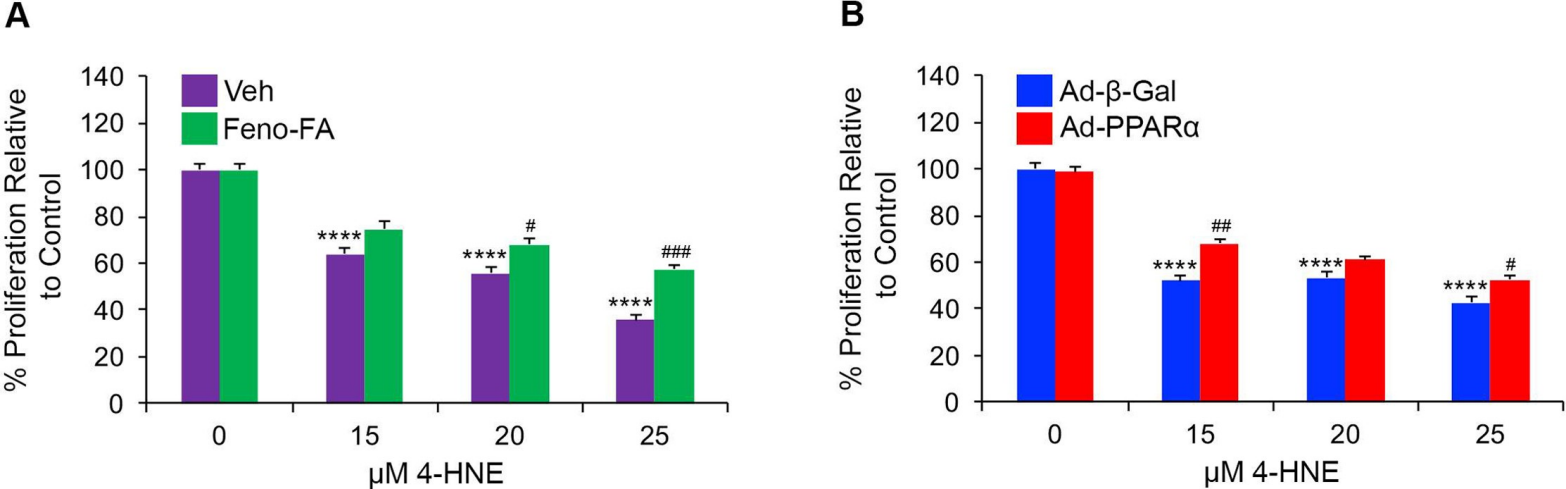
*In vitro* neuroprotective effects of PPARα activation and overexpression. **(A)** MTT assay revealed that cell viability declined in R28 cells treated with the indicated concentrations of 4-Hydroxynonenal (4-HNE) for 24 hours, and was partially restored by treatment with 25 μM fenofibric acid (Feno-FA) relative to vehicle (Veh, DMSO) (n = 6 wells/group). **(B)** MTT assay revealed that cell viability declined in 4-HNE-treated R28 cells, and was partially restored by infection with PPARα-expressing adenovirus (Ad-PPARα, MOI 20) relative to infection with Beta-galactosidase-expressing adenovirus (Ad-β-Gal) (n = 6 wells/group). Data are representative of two independent experiments, and are expressed as mean ± SEM. ****p≤0.0001 relative to (A) Veh or (B) Ad-β-Gal-treated 0 μM HNE groups. #p≤0.05, ##p≤0.01, ###p≤0.001 relative to cells treated with an equal concentration of HNE and (A) Veh or (B) Ad-β-Gal. One-way ANOVA with Tukey’s post-hoc comparison.

To evaluate localized cytoprotective effects of PPARα, we treated cultured R28 rod precursor cells with the diabetic stressor 4-Hydroxynonenal (4-HNE), and used an MTT assay to quantify viability in HNE-stressed cells treated with a PPARα agonist or infected with PPARα-expressing adenovirus. We found that cell viability decreased in 4-HNE-stressed cells (p ≤ 0.0001), and was partially restored by treatment with the PPARα agonist fenofibric acid (p ≤ 0.05) **(Fig 3A)**. Further, infection with PPARα-expressing adenovirus restored cell viability in HNE-stressed cells (p ≤ 0.05) **(Fig 3B)**. These findings suggested that PPARα had a direct neuroprotective effect.

### PPARα restoration of mitochondrial function in diabetic retinas

**Fig 4:**
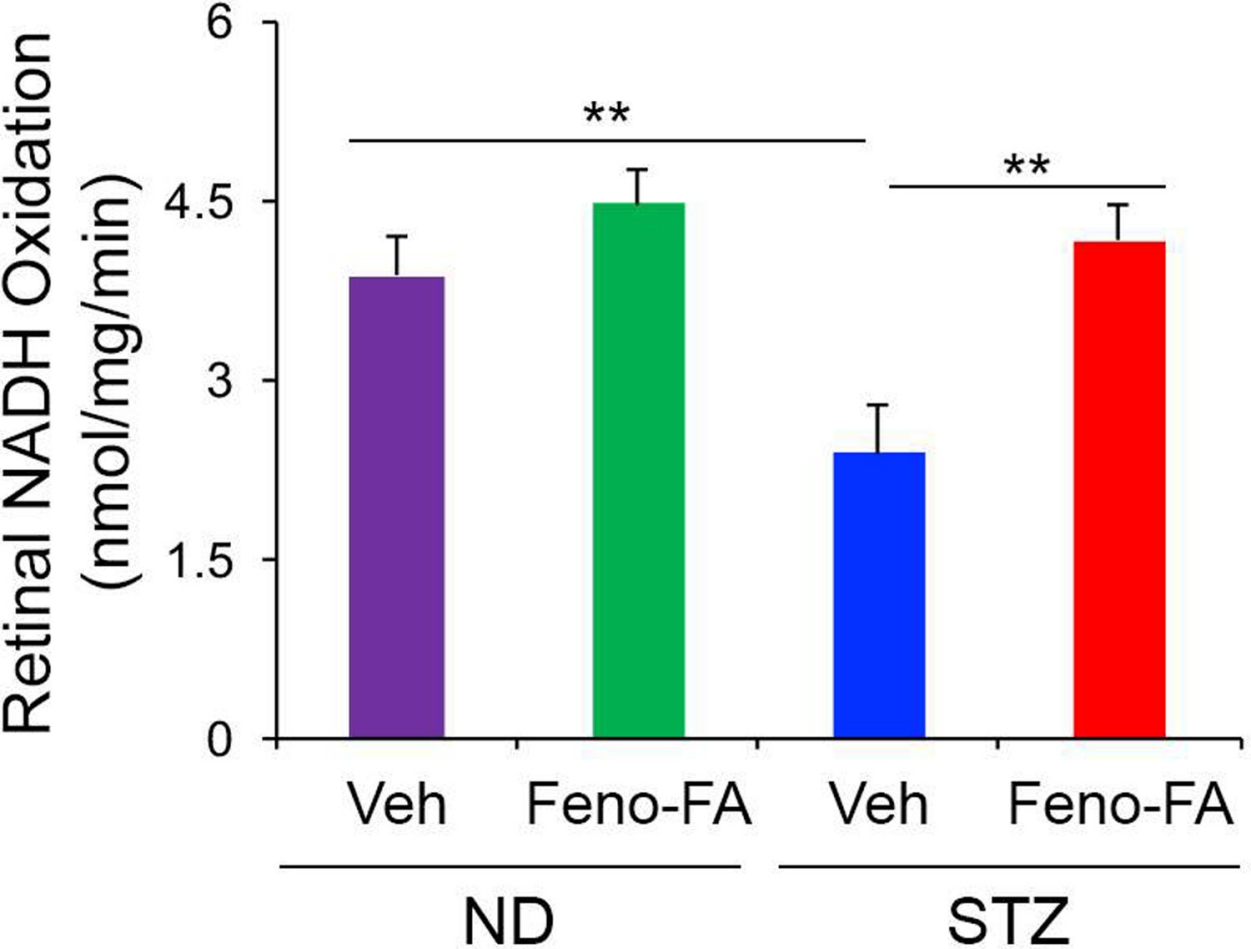
PPARα restoration of retinal NADH-linked respiration in diabetic rats. Retinal NADH oxidation was decreased in Streptozotocin (STZ)-induced type 1 diabetic Sprague Dawley rats at 4 weeks diabetic relative to non-diabetic (ND) rats, and was restored by one week daily injection of fenofibric acid (Feno-FA) relative to Vehicle (Veh, DMSO)-injected diabetic rats. Feno-FA non-significantly increased retinal NADH oxidation in ND rats. (ND-Veh n = 8; ND-Feno-FA n = 7; STZ-Veh n = 4; STZ-Feno-FA n = 6 retinas). Data are expressed as mean ± SEM. *p≤0.05; **p≤0.01, one-way ANOVA with Tukey’s post-hoc comparison.

To determine if PPARα affected mitochondrial function in DR, we measured retinal NADH-linked respiration, which is reflective of electron transport chain activity. We found that in 4-week-diabetic STZ rats, retinal NADH-linked respiration was significantly decreased (p ≤ 0.01), and was partially restored by intraperitoneal injection of PPARα agonist fenofibric acid (p ≤ 0.05) **(Fig 4)**. Fenofibric acid modestly increased retinal NADH-linked respiration in non-diabetic rats, suggesting that PPARα may increase mitochondrial efficiency in normal conditions.

### PPARα restoration of mitochondrial respiration *in vitro*

**Fig 5:**
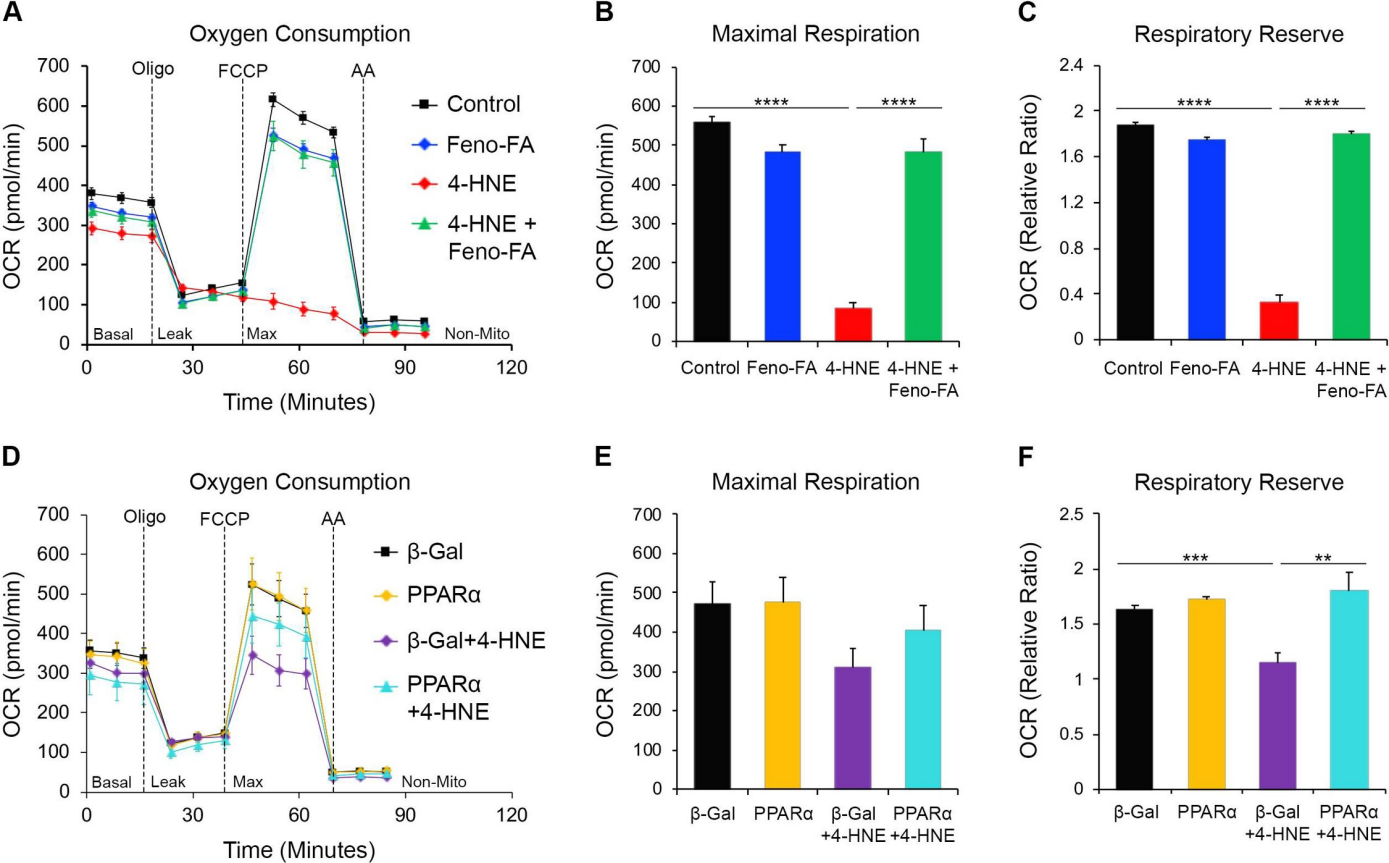
PPARα restores mitochondrial respiration *in vitro*. **(A-C)** Seahorse extracellular flux analysis of R28 cells revealed that (B) maximal respiration and (C) respiratory reserve capacity were decreased by 4 hours exposure to 15 μM 4-Hydroxynonenyl (4-HNE), and restored by a 2-hour pretreatment with 25 μM fenofibric acid (Feno-FA) prior to 4-HNE exposure (n = 5 /group). **(D-F)** Seahorse extracellular flux analysis of adenovirus-infected R28 cells revealed that (E) maximal respiration was non-significantly decreased by 4 hours exposure to 15 μM 4-HNE in cells infected with β-Galactosidase-expressing adenovirus (Ad-β-Gal), and partially restored by infection with PPARα-expressing adenovirus (Ad-PPARα), and (F) respiratory reserve capacity was significantly decreased by 4 hours exposure to 15 μM 4-HNE in Ad-β-Gal infected cells, and restored in 4-HNE-exposed Ad-PPARα-infected cells (β-Gal n = 5, PPARα n = 5, β-Gal + 4-HNE n = 5, PPARα + 4-HNE n = 4). In both experiments, 1 μM oligomycin was injected when indicated in A and D to determine proton leak, 2 μM FCCP was injected as indicated to uncouple (maximize) mitochondrial respiration, and 1 μM antimycin A (AA) was injected at the end of the experiment to arrest mitochondrial respiration. **p≤0.01; ***p≤0.001; ****p≤0.0001, One-way ANOVA with Tukey’s post-hoc comparison.

To evaluate localized effects of PPARα on mitochondrial function, we used Seahorse extracellular flux analysis in 4-HNE-stressed R28 cells pretreated with fenofibric acid or infected with PPARα-expressing adenovirus. We found that maximal respiration significantly decreased in 4-HNE-stressed cells (p≤0.0001), and was restored by pretreatment of cells with fenofibric acid (p ≤ 0.0001) **(Fig 5B).** The respiratory reserve capacity, defined as the difference between basal and maximal (uncoupled) respiration, also significantly declined in 4-HNE-stressed cells (p ≤ 0.0001) and was restored by fenofibric acid pretreatment (p ≤ 0.0001) **(Fig 5C).** In R28 cells infected with β-Gal adenovirus, 4-HNE non-significantly decreased maximal respiration, which was partially restored in 4-HNE-stressed cells infected with Ad-PPARα **(Fig 5E).** Contrastingly, respiratory reserve capacity was significantly decreased by 4-HNE in β-Gal-infected cells (p≤0.001), and was restored by Ad-PPARα infection (p ≤ 0.01) **(Fig 5F).** Viral infection is well-known to affect mitochondrial function in host cells [37], and we suggest that the decreased effects of 4-HNE on mitochondrial function in adenovirus-infected cells may have been due to these effects. Cumulatively, these data suggest that PPARα locally restores mitochondrial function in retinal neuronal cells treated with a diabetic stressor.

### *In vitro* antioxidant effects of PPARα activation and overexpression

**Fig 6:**
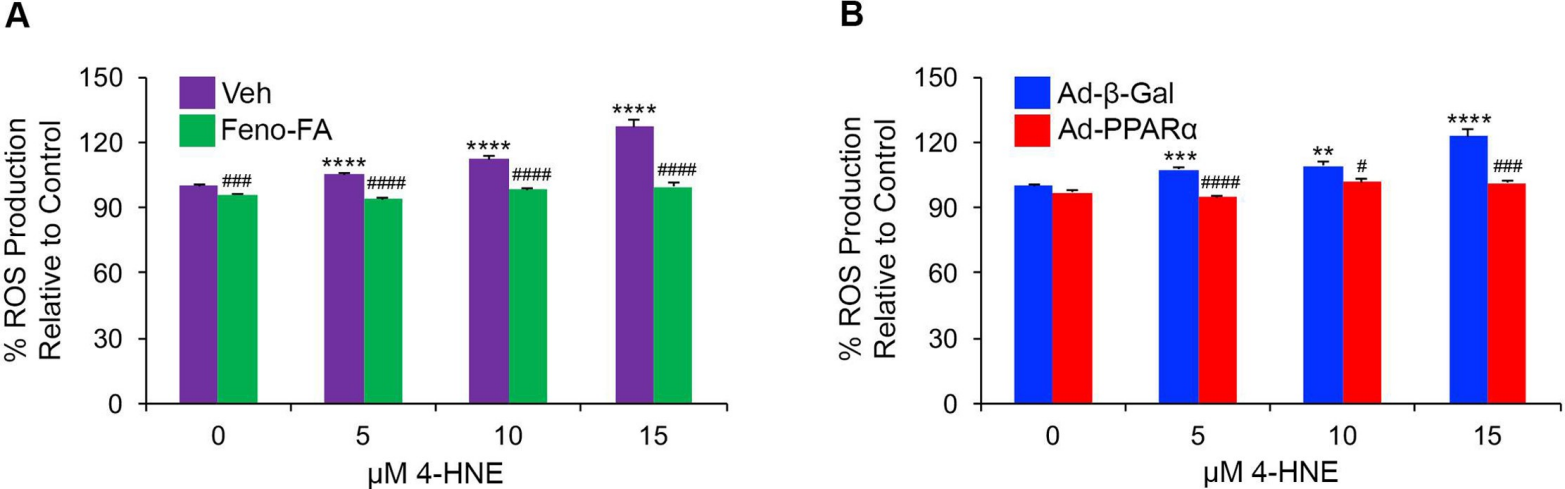
*In vitro* antioxidant effects of PPARα activation and overexpression. **(A)** H_2_DCFDA ROS detection assay revealed that ROS production was increased in R28 cells treated with the indicated concentrations of 4-Hydroxynonenal (4-HNE) for one hour, and was decreased to baseline by pretreatment with 25 μM fenofibric acid (Feno-FA) for 7 hours relative to vehicle (Veh, DMSO)-treated cells. Feno-FA modestly decreased ROS production in cells treated with 0 μM HNE (n = 9 wells/group). **(B)** ROS assay revealed that ROS production was increased in 4-HNE-treated R28 cells, and was decreased by infection with PPARα-expressing adenovirus (Ad-PPARα, MOI 20) relative to infection with Beta-galactosidase-expressing adenovirus (Ad-β-Gal). Infection with Ad-PPARα non-significantly decreased ROS production in cells treated with 0 μM HNE (n = 6 wells/group). Data are representative of 2–3 independent experiments, and are expressed as mean ± SEM. **p≤0.01; ***p≤0.001; ****p≤0.0001 relative to (A) Veh or (B) Ad-β-Gal-treated 0 μM HNE groups. #p≤0.05; ##p≤0.01; ###p≤0.001; ####p≤0.0001 relative to cells treated with an equal concentration of HNE and (A) Veh or (B) Ad-β-Gal. One-way ANOVA with Tukey’s post-hoc comparison.

To evaluate localized antioxidant effects of PPARα, we measured ROS production in 4-HNE-stressed R28 cells pretreated with fenofibric acid or infected with PPARα-expressing adenovirus. We found that ROS production increased in a dose-dependent manner with one hour of 4-HNE treatment (p ≤ 0.01), and was decreased by pretreatment of cells with fenofibric acid (p ≤ 0.001) **(Fig 6A)**. Further, we found that infection with PPARα-expressing adenovirus decreased ROS production in 4-HNE-stressed cells (p ≤ 0.05) **(Fig 6B)**. Both fenofibric acid and PPARα overexpression decreased ROS production in control conditions, suggesting that PPARα may decrease mitochondrial ROS production in a basal state.

### Increased oxidative stress in diabetic *Pparα*^*-/-*^ mice

**Fig 7:**
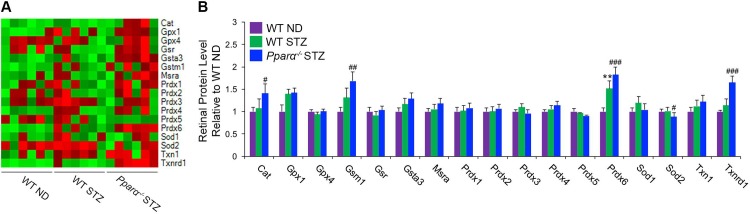
Increased oxidative stress markers in *Pparα*^*-/-*^ diabetic retinas. **(A)** Heatmap representation of quantitative proteomic analysis of wild-type (WT) non-diabetic (ND) retinas, WT streptozotocin (STZ)-induced diabetic retinas and *Pparα*^*-/-*^ diabetic retinas at 28 weeks diabetic. **(B)** Relative retinal protein levels of antioxidant enzymes Gsm1, Prdx6 and Txnrd1 were increased in WT diabetic mice and further increased in *Pparα*^*-/-*^ diabetic retinas. Cat was increased in *Pparα*^*-/-*^ diabetic retinas relative to WT diabetic retinas. (n = 6 mice/group). *p≤0.05; **p≤0.01relative to WT ND group. #p≤0.05; ###p≤0.001 relative to WT diabetic group. One-way ANOVA with Tukey’s post-hoc comparison.

To evaluate potential *in vivo* antioxidant effects of PPARα, we used a quantitative multiplex proteomics approach to measure retinal levels of antioxidant enzymes in wild-type and *Pparα*^*-/-*^ mice with STZ-induced diabetes. We found that at 28 weeks diabetic, retinal levels of antioxidant enzymes Gsm1, Prdx6 and Txnrd1 were increased in WT STZ mice, and further increased in *Pparα*^*-/-*^ STZ mice (p ≤ 0.05) **(Fig 7)**. Full gene names are shown in **Table 1.** Cat was significantly increased in *Pparα*^*-/-*^ diabetic mice relative to wild-type diabetic mice (p ≤ 0.05). These data suggest that oxidative stress may be exacerbated by PPARα ablation in DR.

**Table 1:** Full names of genes presented in Fig 7.

Gene Symbol	Full Name
Cat	Catalase
Gpx1	Glutathione Peroxidase 1
Gpx4	Glutathione Peroxidase 4
Gsr	Glutathione-Disulfide Reductase
Gsta3	Glutathione-S-Transferase Apha 3
Gstm1	Glutathione-S-Transferase M1
Msra	Methionine Sulfide Reductase A
Prdx1	Peroxidase 1
Prdx2	Peroxidase 2
Prdx3	Peroxidase 3
Prdx4	Peroxidase 4
Prdx5	Peroxidase 5
Prdx6	Peroxidase 6
Sod1	Superoxide Dismutase 1
Sod2	Superoxide Dismutase 2
Txn1	Thioredoxin 1
Txnrd1	Thioredoxin Reductase 1

## Discussion

In the present study, we demonstrated for the first time that PPARα is neuroprotective in retinopathy of type 1 diabetes, and is therefore a potential therapeutic for DR in type 1 diabetic patients. PPARα is a well-known regulator of energy metabolism and mitochondrial function [17], and we identified here that PPARα supported mitochondrial function and decreased ROS production in DR, which would alleviate primary energetic shortages, mitochondrially mediated apoptosis and cellular oxidative damage that contribute to neurodegeneration in DR [13].

DR is considered to be a microvascular complication of diabetes, and microvascular lesions are the primary diagnostic criteria and therapeutic targets for DR clinically [1]. However, neurodegeneration begins prior to the appearance of overt microvascular lesions, and likely contributes to microvascular dysfunction [2]. Here we find that PPARα is neuroprotective in DR **(Figs 1–3)**, which may be responsible in part for therapeutic effects of the PPARα agonist fenofibrate identified in clinical trials [4, 5]. Further, diabetes-induced PPARα down-regulation in the retina may contribute to retinal neurodegeneration in DR.

We found that PPARα activation with fenofibrate restored retinal DNA fragmentation (apoptosis) to that of non-diabetic animals **(Fig 1C)**, but that functional recovery of visual acuity as measured by the optokinetic reflex was only partial **(Fig 1A and 1B)**. We postulate that this seeming discrepancy could be because factors other than neurodegeneration contribute to optokinetic reflex in DR, and/or because mechanisms unrelated to PPARα affect long-term visual function. The optokinetic reflex declines in DR due to both neurodegeneration and unrelated pathologies such as increased cataract formation in DR and impaired reaction time in diabetic animals [21]. To our knowledge PPARα does not affect cataract formation or reaction time in diabetes, so the readout of neuroprotection in fenofibrate-treated diabetic animals may be precluded in part by these unrelated pathologies. Further, DR is a multifactorial disease, and it is likely that long-term loss of neuronal function, as assessed by optokinetic tracking, is due in part to mechanisms unaffected by PPARα, for example endoplasmic reticulum stress or advanced glycation end products.

The retina is the most metabolically demanding tissue of the body, and therefore must produce abundant ATP [18]. Photoreceptor mitochondria thus function at full capacity with a limited respiratory reserve in order to meet energetic demands [12]. Because of this adaptation, the retina is a highly oxidative tissue, and is more sensitive to mitochondrial dysfunction and metabolic derangements than many other tissues. Retinal mitochondrial dysfunction begins early in diabetes, and is known to contribute to DR pathology [15]. Mitochondrial dysfunction increases production of neurotoxic ROS, and is also likely to result in primary energetic deficits that are detrimental to retinal neurons [15].

We demonstrate in the present study that retinal NADH-linked respiration, reflective of electron transport chain activity, is decreased in DR, and is partially restored by PPARα activation **(Fig 4)**. PPARα is a known regulator of lipid and glucose metabolism, and also of mitochondrial homeostasis, so likely improves energy efficiency through several mechanisms [17, 38]. These findings suggest that PPARα alleviates primary energetic deficits that contribute to neurodegeneration in DR. Further, mitochondria produce more ROS when the NADH/NAD^+^ ratio is increased and/or ATP synthesis is impaired, so restoring NADH oxidation and alleviating mitochondrial dysfunction is also likely to decrease ROS production [14], decreasing cellular and mitochondrial oxidative damage.

Because the retina is a highly oxidative tissue, it may be more prone to increased ROS production and oxidative stress in diabetic conditions. We demonstrate here that PPARα decreases ROS production *in vitro* **(Fig 6)**, and that diabetic *Pparα*^*-/-*^ mice have increased retinal levels of antioxidant enzymes, suggestive of increased oxidative stress **(Fig 7)**. Together, these findings demonstrate a potent antioxidant effect for PPARα in retinopathy of type 1 diabetes, which may be modulated in part through improved mitochondrial dysfunction and subsequent decreases in ROS production.

Increased mitochondrial ROS production or decreased antioxidant capacity exacerbate mitochondrial DNA damage and dysfunction in DR [13]. ROS released into the cytoplasm are highly reactive and oxidize cellular macromolecules. In particular, retinal neurons are lipid-rich, and lipids are highly susceptible to oxidative damage by intracellular ROS. Oxidized lipids are neurotoxic, and are known to contribute to neurodegeneration in many disease states, including DR [39]. By alleviating oxidative stress in DR, PPARα is likely to confer neuroprotection through several mechanisms, including improved mitochondrial function and decreased oxidation of intracellular macromolecules.

We revealed here that PPARα is neuroprotective in retinopathy of type 1 diabetes, and that these effects may be mediated in part through improved mitochondrial efficiency and subsequent decreases in mitochondrial ROS production. These findings have three major implications: (i) PPARα is neuroprotective in retinopathy of type 1 diabetes; (ii) PPARα increases mitochondrial efficiency and alleviates oxidative stress under diabetic conditions; and (iii) PPARα is a putative therapeutic target for retinopathy and other neurodegenerative conditions of type 1 diabetes. This work advances basic understanding of the metabolic basis of DR, and has significant translational potential.

## Supporting information

S1 Table Weight (g) of Brown Norway rats was measured 72 hours after STZ injection and monthly thereafter. Shown are mean ± SEM. ND, Non-Diabetic; Ctrl, Control; Feno, Fenofibrate; STZ Streptozotocin-diabetic.
(DOCX)

S2 Table Blood glucose (mg/dL) of Brown Norway rats was measured 72 hours after STZ injection and monthly thereafter. Shown are mean ± SEM. ND, Non-Diabetic; Ctrl, Control; Feno, Fenofibrate; STZ Streptozotocin-diabetic.
(DOCX)

S3 Table Weight (g) of Sprague Dawley rats was measured 72 hours after STZ injection and weekly thereafter. Shown are mean ± SEM. ND, Non-Diabetic; Ctrl, Control; Feno, Fenofibric Acid; STZ Streptozotocin-diabetic.
(DOCX)

S4 Table Blood glucose (mg/dL) of Sprague Dawley rats was measured 72 hours after STZ injection and weekly thereafter. Shown are mean ± SEM. ND, Non-Diabetic; Ctrl, Control; Feno, Fenofibric Acid; STZ Streptozotocin-diabetic.
(DOCX)

S5 Table Weight (g) of mice was measured 5 days after STZ injection and monthly thereafter. Shown are mean ± SEM. WT, Wild-type; ND, Non-Diabetic; STZ Streptozotocin-diabetic.
(DOCX)

S6 Table Blood glucose (mg/dL) of mice was measured 5 days after STZ injection and monthly thereafter. Shown are mean ± SEM. WT, Wild-type; ND, Non-Diabetic; STZ Streptozotocin-diabetic.
(DOCX)
