# Supplementary material for: Neuroprotective effects of PPARα in retinopathy of type 1 diabetes
Source: PLoS One. 2019 Feb 4;14(2):e0208399. doi: 10.1371/journal.pone.0208399 (PMC6361421; doi:10.1371/journal.pone.0208399)
Supplement: S1 Table — Shown are mean ± SEM. ND, Non-Diabetic; Ctrl, Control; Feno, Fenofibrate; STZ Streptozotocin-diabetic. (DOCX) [file pone.0208399.s001.docx]

**Supplementary Table 1: Weight of Brown Norway STZ Rats**

|  | Group | | | |
| --- | --- | --- | --- | --- |
| Duration Diabetes | ND Ctrl | ND Feno | STZ Ctrl | STZ Feno |
| 72 hours | 150.5 ± 2.79 | 145.6 ± 5.09 | 148.9 ± 3.89 | 153.1 ± 4.03 |
| 4 weeks | 168.2 ± 2.52 | 170.1 ± 2.69 | 151.2 ± 3.75 | 157.5 ± 2.90 |
| 8 weeks | 181.3 ± 2.85 | 182.1 ± 3.25 | 155.4 ± 4.09 | 165.4 ± 3.98 |
| 12 weeks | 192.0 ± 3.24 | 190.6 ± 2.33 | 158.6 ± 4.91 | 169.6 ± 3.65 |

**Supplementary Table 1:**  Weight (g) of Brown Norway rats was measured 72 hours after STZ injection and monthly thereafter. Shown are mean ± SEM. ND, Non-Diabetic; Ctrl, Control; Feno, Fenofibrate; STZ Streptozotocin-diabetic.
