# Supplementary material for: Neuroprotective effects of PPARα in retinopathy of type 1 diabetes
Source: PLoS One. 2019 Feb 4;14(2):e0208399. doi: 10.1371/journal.pone.0208399 (PMC6361421; doi:10.1371/journal.pone.0208399)
Supplement: S2 Table — Shown are mean ± SEM. ND, Non-Diabetic; Ctrl, Control; Feno, Fenofibrate; STZ Streptozotocin-diabetic. (DOCX) [file pone.0208399.s002.docx]

**Supplementary Table 2: Blood glucose of Brown Norway STZ Rats**

| Duration Diabetes | Group | | | |
| --- | --- | --- | --- | --- |
|  | ND Ctrl | ND Feno | STZ Ctrl | STZ Feno |
| 72 hours | 119.2 ± 3.22 | 123.2 ± 2.41 | 591.8 ± 7.72 | 553.1 ± 26.96 |
| 4 weeks | 124.6 ± 4.02 | 115.4 ± 3.92 | 585.9 ± 10.02 | 589.7 ± 18.78 |
| 8 weeks | 118.7 ± 3.75 | 121.3 ± 2.08 | 579.8 ± 21.01 | 575.8 ± 15.23 |
| 12 weeks | 106.7 ± 4.20 | 102.4 ± 2.41 | 531.75 ± 32.64 | 528.4 ± 18.25 |

**Supplementary Table 2:**  Blood glucose (mg/dL) of Brown Norway rats was measured 72 hours after STZ injection and monthly thereafter. Shown are mean ± SEM. ND, Non-Diabetic; Ctrl, Control; Feno, Fenofibrate; STZ Streptozotocin-diabetic.
