# Supplementary material for: Neuroprotective effects of PPARα in retinopathy of type 1 diabetes
Source: PLoS One. 2019 Feb 4;14(2):e0208399. doi: 10.1371/journal.pone.0208399 (PMC6361421; doi:10.1371/journal.pone.0208399)
Supplement: S3 Table — Shown are mean ± SEM. ND, Non-Diabetic; Ctrl, Control; Feno, Fenofibric Acid; STZ Streptozotocin-diabetic. (DOCX) [file pone.0208399.s003.docx]

**Supplementary Table 3: Weight of Sprague Dawley STZ Rats**

| Duration Diabetes | Group | | | |
| --- | --- | --- | --- | --- |
|  | ND Ctrl | ND Feno | STZ Ctrl | STZ Feno |
| 72 hours | 219.2 ± 12.02 | 218.6 ± 10.12 | 183.3 ± 31.32 | 175.6 ± 16.51 |
| 1 week | 259.4 ± 13.05 | 262.2 ± 11.12 | 191.4 ± 33.68 | 184.6 ± 29.93 |
| 2 weeks | 296.4 ± 18.88 | 299.3 ± 11.20 | 173.7 ± 20.24 | 179.4 ± 36.79 |
| 3 weeks | 317.2 ± 18.27 | 372.1 ± 16.22 | 176.9 ± 25.04 | 185.4 ± 41.00 |
| 4 weeks | 334.2 ± 18.19 | 349.2 ± 15.83 | 171.1 ± 25.22 | 182.0 ± 37.60 |

**Supplementary Table 3:**  Weight (g) of Sprague Dawley rats was measured 72 hours after STZ injection and weekly thereafter. Shown are mean ± SEM. ND, Non-Diabetic; Ctrl, Control; Feno, Fenofibric Acid; STZ Streptozotocin-diabetic.
