# Supplementary material for: Neuroprotective effects of PPARα in retinopathy of type 1 diabetes
Source: PLoS One. 2019 Feb 4;14(2):e0208399. doi: 10.1371/journal.pone.0208399 (PMC6361421; doi:10.1371/journal.pone.0208399)
Supplement: S4 Table — Shown are mean ± SEM. ND, Non-Diabetic; Ctrl, Control; Feno, Fenofibric Acid; STZ Streptozotocin-diabetic. (DOCX) [file pone.0208399.s004.docx]

**Supplementary Table 4: Blood Glucose of Sprague Dawley STZ Rats**

| Duration Diabetes | Group | | | |
| --- | --- | --- | --- | --- |
|  | ND Ctrl | ND Feno | STZ Ctrl | STZ Feno |
| 72 hours | 116.8 ± 6.37 | 126.2 ± 12.46 | 425.8 ± 62.99 | 441.0 ± 87.89 |
| 1 week | 118.6 ± 6.17 | 124.2 ± 19.97 | 528.1 ± 69.57 | 510.4 ± 73.73 |
| 2 weeks | 123.2 ± 12.62 | 119.0 ± 7.48 | 522.4 ± 66.21 | 496.6 ± 63.30 |
| 3 weeks | 106.6 ± 6.87 | 115.2 ± 11.36 | 474.0 ± 57.40 | 463.3 ± 59.55 |
| 4 weeks | 110.1 ± 8.87 | 160.7 ± 33.19 | 547.7 ± 52.40 | 494.9 ± 64.95 |

**Supplementary Table 4:**  Blood glucose (mg/dL) of Sprague Dawley rats was measured 72 hours after STZ injection and weekly thereafter. Shown are mean ± SEM. ND, Non-Diabetic; Ctrl, Control; Feno, Fenofibric Acid; STZ Streptozotocin-diabetic.
