# Supplementary material for: Neuroprotective effects of PPARα in retinopathy of type 1 diabetes
Source: PLoS One. 2019 Feb 4;14(2):e0208399. doi: 10.1371/journal.pone.0208399 (PMC6361421; doi:10.1371/journal.pone.0208399)
Supplement: S5 Table — Shown are mean ± SEM. WT, Wild-type; ND, Non-Diabetic; STZ Streptozotocin-diabetic. (DOCX) [file pone.0208399.s005.docx]

**Supplementary Table 5: Weight of STZ Mice**

| Duration Diabetes | Group | | | |
| --- | --- | --- | --- | --- |
|  | WT ND | WT STZ | *Pparα^-/-^* ND | *Pparα^-/-^* STZ |
| 5 days | 26.5 ± 0.65 | 23.4 ± 0.95 | 30.8 ± 0.25 | 22.7 ± 1.14 |
| 4 weeks | 26.0 ± 0.64 | 23.4 ± 0.60 | 30.2 ± 0.25 | 23.5 ± 1.25 |
| 8 weeks | 26.3 ± 0.81 | 24.3 ± 0.82 | 28.7 ± 0.56 | 24.2 ± 1.28 |
| 12 weeks | 27.9 ± 0.78 | 24.5 ± 0.79 | 29.6 ± 0.75 | 22.9 ± 1.40 |
| 16 weeks | 28.7 ± 0.85 | 25.2 + 1.26 | 30.7 ± 0.83 | 22.6 ± 1.57 |
| 20 weeks | 30.14 ± 0.79 | 23.9 ± 1.54 | 31.7 ± 0.71 | 22.5 ± 2.15 |
| 24 weeks | 30.42 ± 0.81 | 23.8 ± 2.08 | 31.4 ± 0.56 | 22.6 ± 2.49 |

**Supplementary Table 5:**  Weight (g) of mice was measured 5 days after STZ injection and monthly thereafter. Shown are mean ± SEM. WT, Wild-type; ND, Non-Diabetic; STZ Streptozotocin-diabetic.
