# Supplementary material for: Neuroprotective effects of PPARα in retinopathy of type 1 diabetes
Source: PLoS One. 2019 Feb 4;14(2):e0208399. doi: 10.1371/journal.pone.0208399 (PMC6361421; doi:10.1371/journal.pone.0208399)
Supplement: S6 Table — Shown are mean ± SEM. WT, Wild-type; ND, Non-Diabetic; STZ Streptozotocin-diabetic. (DOCX) [file pone.0208399.s006.docx]

**Supplementary Table 6: Blood Glucose of STZ Mice**

| Duration Diabetes | Group | | | |
| --- | --- | --- | --- | --- |
|  | WT ND | WT STZ | *Pparα^-/-^* ND | *Pparα^-/-^* STZ |
| 5 days | 164.2 ± 18.11 | 427.3 ± 29.92 | 175.8 ± 23.94 | 446.5 ± 32.98 |
| 4 weeks | 166.0 ± 4.38 | 375.0 ± 32.68 | 136.8 ± 9.56 | 380.8 ± 17.60 |
| 8 weeks | 126.4 ± 6.99 | 404.5 ± 28.58 | 121.8 ± 11.66 | 445.6 ± 34.31 |
| 12 weeks | 129.8 ± 9.05 | 409.4 ± 62.98 | 128.2 ± 9.21 | 510.3 ± 30.22 |
| 16 weeks | 128.4 ± 4.95 | 412.6 ± 56.54 | 122.3 ± 8.63 | 484.0 ± 44.72 |
| 20 weeks | 121.7 ± 9.51 | 421.8 ± 76.91 | 115.0 ± 8.14 | 479.8 ± 44.68 |
| 24 weeks | 136.6 ± 5.62 | 446.5 ± 81.50 | 133.0 ± 9.46 | 497.3 ± 46.45 |

**Supplementary Table 6:**  Blood glucose (mg/dL) of mice was measured 5 days after STZ injection and monthly thereafter. Shown are mean ± SEM. WT, Wild-type; ND, Non-Diabetic; STZ Streptozotocin-diabetic.
